# Evaluating the Survival Benefit Following Ovarian Function Suppression in Premenopausal Patients with Hormone Receptor Positive Early Breast Cancer

**DOI:** 10.1038/srep26627

**Published:** 2016-05-27

**Authors:** Lin Qiu, Fangmeng Fu, Meng Huang, Yuxiang Lin, Yazhen chen, Minyan Chen, Chuan Wang

**Affiliations:** 1 Union Hospital School, Fujian Medical University, Fuzhou, China; 2 Department of Breast Surgery, Affiliated Union Hospital of Fujian Medical, University, Fuzhou, China; 3 Fujian Center for Disease Control and Prevention, China

## Abstract

There are divergent opinions regarding the use of ovarian function suppression or ablation (hereafter, OFS) in hormone receptor positive early breast cancer patients. In order to clarify the survival benefit of OFS, a meta-analysis was performed. The result is that use of OFS was more effective than no OFS on DFS (the pooled relative risk (pRR) = 0.86; 95% CI: 0.75–0.96) and on OS (pRR = 0.79; 95% CI: 0.70–0.89). In subgroup analysis, we found that increased DFS was positively associated with patients who had received chemotherapy (pRR = 0.85; 95% CI: 0.74–0.96), who were lymph node negative (pRR = 0.74; 95% CI: 0.61–0.91) and were less than 40 years old (pRR = 0.71; 95% CI: 0.59–0.83). There was a significant difference in OS between the groups receiving chemotherapy (pRR = 0.73; 95% CI: 0.58–0.89) or for patients less than 40 years old (pRR = 0.52; 95% CI: 0.18–0.87). The use of OFS also produces statistical differences in the occurrence of the side-effects; severe hot flashes (pRR = 2.32; 95% CI: 1.36–3.97), and hypertension (pRR = 1.54; 95% CI: 1.12–2.12). In general, OFS should be considered as one treatment for hormone receptor positive premenopausal early breast cancer patients who have received chemotherapy and are less than 40 years old. We also should pay attention to the side-effects and weigh the advantages and disadvantages before deciding on using OFS.

It has been over a century since Beatson demonstrated that oophorectomy was effective for treating advanced breast cancer. Since then, endocrine therapies have become firmly established for managing all stages of breast cancer^1^.

The choice of the endocrine agent for breast cancer depends on the menopausal status of the patient, the stage of disease, prognostic factors, and the toxicity profile of the agent. Endocrine therapies are typically given sequentially with the least toxic therapy given first. Tamoxifen is considered a first-line endocrine therapy for all stages of breast cancer^1^. Subsequent to its proven efficacy for advanced disease, the drug was employed as adjuvant treatment for the management of early operable breast cancer and it is now the most widely used hormonal therapy for treating the disease. More than 3 million breast cancer patients have received tamoxifen^2^ for greater than 5.8 million patient-years.

Tamoxifen has also become the drug of choice for the endocrine treatment of advanced breast cancer in postmenopausal women who are considered likely to respond to endocrine treatment. In the adjuvant setting, tamoxifen provides significant clinical benefits in patients with early-stage breast cancer, prolonging survival^3^ and it reduces the incidence of new contralateral breast tumors^4^,^5^. A significant number of patients, however, still experience disease recurrence or progression during tamoxifen therapy, and despite a good overall tolerability profile 4,6 the long-term use is associated with a two- to three-fold increase in the risk of developing endometrial cancer^5^.

In 2001, the initial results of the Anastrozole, Tamoxifen Alone or in Combination trial revealed a statistically significant improvement in disease-free survival for postmenopausal women taking initial anastrozole compared with initial tamoxifen as adjuvant therapy for hormone receptor-positive early breast cancer^7^. These findings led to the subsequent United States Food and Drug Administration approval of anastrozole as adjuvant therapy for postmenopausal women with hormone receptor positive early breast cancer, and widespread adoption of the practice of prescribing initial anastrozole therapy for many women with early breast cancer. Subsequent large trials have confirmed its role in postmenopausal women with hormone receptor-positive early-stage breast cancer^8^,^9^,^10^.

Before menopause, up to 90% of hormones are produced by the ovaries in women^11^. Thus, ovarian ablation has become an important part of endocrine therapy and has been widely accepted in treatment of breast cancer since 1896^12^. However, with the development of adjuvant therapy for breast cancer, there has been less emphasis on ovarian ablation. With the introduction of medical ovarian ablation using luteinizing hormone releasing hormone-agonists (LHRH-agonists), ovarian ablation with LHRH-agonists has attracted increasing attention due to its ability to reversibly suppress estrogen secretion by the ovary. Some studies have shown that ovarian suppression or ablation improves the survival of premenopausal breast cancer patients who are hormone receptor-positive and have received adjuvant therapy or palliative care^13^,^14^.

The mechanism of bilateral oophorectomy or bilateral ovarian irradiation reduces ovarian function and results in a decline in oestradiol to post-menopausal concentrations by directly acting on the ovaries. However, LHRH-agonists act on the hypothalamic-pituitary axis, achieving ovarian suppression by LHRH receptor down-regulation^15^ (Fig. 1).

The best method for performing ovarian suppression or ablation remains controversial^13^,^16^,^17^. As current evidence does not adequately resolve questions of the use of OFS in hormone receptor positive early breast cancer patients, the present systematic review and meta-analysis was conducted with the aim of assessing the efficacy of OFS in premenopausal hormone receptor positive early breast cancer patients.

## Results

A total of 1,317 publications were retrieved from the electronic data-bases. After duplicates were removed, 1,200 records were screened for eligibility. We excluded a further 1,054 on the basis of title or abstract, and the remaining 146 study reports were retrieved for more detailed evaluation (Fig. 2). Overall, 11 published studies^16^,^18^,^19^,^20^,^21^,^22^,^23^,^24^,^25^,^26^,^27^ fulfilled the criteria for eligibility and were included in the review (Table 1).

### Characteristics of studies included in the meta-analysis

All included studies were randomized prospective cohort studies, and the total number of participants involved in these randomized controlled trials was 12,292. The patients of all trials were of a premenopausal status; for three studies the status was determined after chemotherapy. Even though hormone receptors of some patients are negative in the included population, we choose the patients who were positive for hormone receptors for our analysis. There were only five studies analyzing the survival benefit of the number of lymph nodes that transitioned from negative to positive. The majority of patients were treated with chemotherapy, although some did not receive chemotherapy in the E-3193 and SOFT studies. Five studies analyzed the difference between patients who were more than 40 years old and less than 40 years old. Only four trials had the relevant data about side-effects.

### Overall survival

The present systematic review assessed the efficacy of OFS in premenopausal early breast cancer patients by pooling together studies that enrolled patients with positive hormone receptors. This approach has allowed, for the first time, consideration of all the evidence based on the beneficial and harmful effects of OFS in early breast cancer patients. According to the data which were abstracted from articles included, we drew a total forest plot which is designed to demonstrate data distribution. The pooled relative risk (pRR) of DFS is 0.86 which is less than 1, and 95% confidence interval (95% CI) is 0.75–0.96 which does not contain 1. This indicated that OFS could improve DFS for premenopausal women with hormone receptor positive early breast cancer. In order to prevent the possibility of publication offset, we drew a funnel plot which had a basic symmetrical distribution, indicating no publication offset. In the same way, we determined total OS of OFS, whose pRR is 0.79 and the 95% CI is 0.70–0.89. This also showed that OFS could improve the OS for premenopausal women with hormone receptor positive early breast cancer (Fig. 3).

### Subgroup analysis

In order to further study suitable groups of OFS patients, we carried out four subgroup analyses including lymph positive or negative, chemotherapy or no chemotherapy, less than 40 years old or not, and side-effect after OFS.

**Figure 1:**
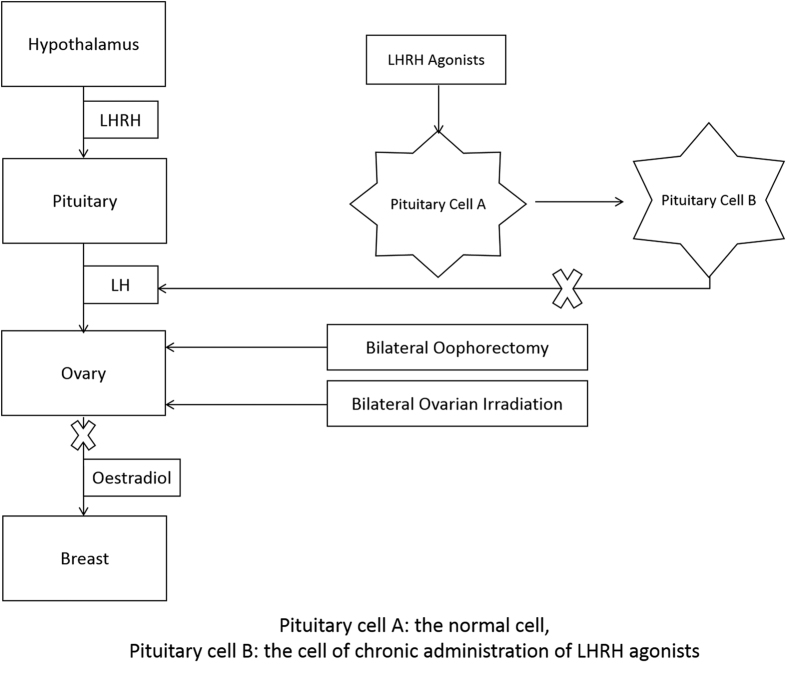


**Figure 2:**
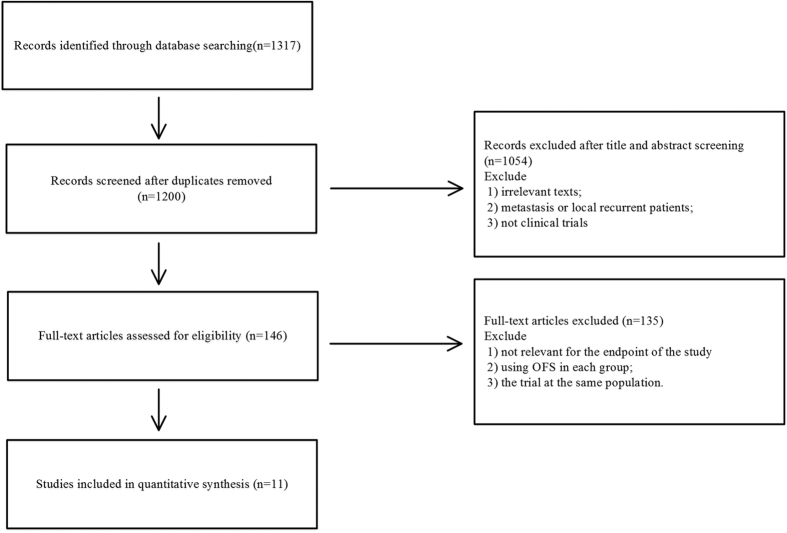


- Subgroup analysis based on the chemotherapy group. We also analyzed the difference between receiving OFS and no OFS when both the experimental and control groups had received chemotherapy. There are eight studies including in this subgroup. Through statistical analysis and forest plot analysis, we see that with ovarian function suppression there is 0.85 times the risk of death compared to no OFS (pRR = 0.85, 95% CI: 0.74–0.96). For OS, the pRR is 0.73 (pRR = 0.73, 95% CI: 0.58–0.89). This illustrates that women with premenopausal hormone receptor positive early breast cancer receiving chemotherapy could obtain survival benefits from OFS (Fig. 4).
- Subgroup analysis based on no chemotherapy group. For patients with no chemotherapy Fig. 4 shows that the DFS in the group receiving OFS is 0.85 times that of the control group (pRR = 0.85, 95% CI: 0.54–1.16). Figure 4 shows that the OS in the experimental group is 0.85 times that of the control group (pRR = 0.85, 95% CI: 0.14–1.56). Because of the 95% confident interval includes 1, there is no statistical significance in the difference, and it is unknown if patients will obtain survival benefits through OFS (Fig. 4).
- Subgroup analysis based on lymph node positive or negative status. There were three studies that included lymph node involvement, showing that patients who go through OFS have 0.97 times the risk of death compared to those who do not (pRR = 0.97, 95% CI: 0.86–1.09). The pRR result from three articles with patients with negative lymph nodes is 0.74 (95% CI: 0.61–0.91). In accordance with the statistical results, we reached the conclusion that patients with negative lymph nodes could have an improved disease free survival by OFS, but the lymph node positive patients did not benefit from OFS. However, when the lymph node positive and negative patients were grouped, the OS did not improve with OFS (Fig. 5).
- Subgroup analysis based on age. We found that the age of patients, older or younger than 40 years, has an effect on the heterogeneity observed in the results. There were five studies that included results from patients more than 40 years old. Figure 6 shows that the experimental group has 0.88 times the risk of death or recurrent disease compared to the control group in patients more than 40 years old, and the 95% confident interval is 0.71 to 1.06. (pRR = 0.88, 95% CI: 0.71–1.06). Since the 95% CI includes 1, this suggests that this group did not obtain significant survival benefits from OFS. However, in the less than 40 years old group, the pRR is 0.71 and the 95% CI is 0.59–0.83 (pRR = 0.71, 95% CI: 0.59–0.83). Considering the two results, we conclude that younger patients may obtain better survival benefits than older patients. Because of the availability of the data, we could only analyze data from three studies including the OS of patients more than 40 years old receiving OFS and OS from three studies of patients less than 40 years old. From Fig. 6 we conclude the same result, that patients less than 40 year old could achieve a survival benefit from OFS.
- Subgroup analysis based on side-effect of OFS. The subgroup analysis of side-effects showed a statistically significant effect of OFS over no OFS in participants with early breast cancer: for hot flashes (pRR = 1.54, 95% CI: 1.19 to 2.01; pRR:2.32, 95% CI:1.36 to 3.97 on more than grade 3) and for hypertension (pRR = 1.53, 95% CI: 1.17 to 1.99). Treatment with OFS and no OFS made no difference in terms of weight gain (2 trials included, pRR = 1.26, 95% CI: 0.93 to 1.69), diabetes (2 trials included, pRR = 1.41, 95% CI: 0.90 to 1.69) and grade 3 or higher toxicities from hypertension (pRR = 2.10, 95% CI: 0.87 to 5.09), weight gain (pRR = .1.10, 95% CI: 0.78 to 1.55) and diabetes (pRR = 1.85, 95% CI: 0.36 to 9.63) (Supplement Figs 1 and 2).

### Publication bias

**Figure 3:**
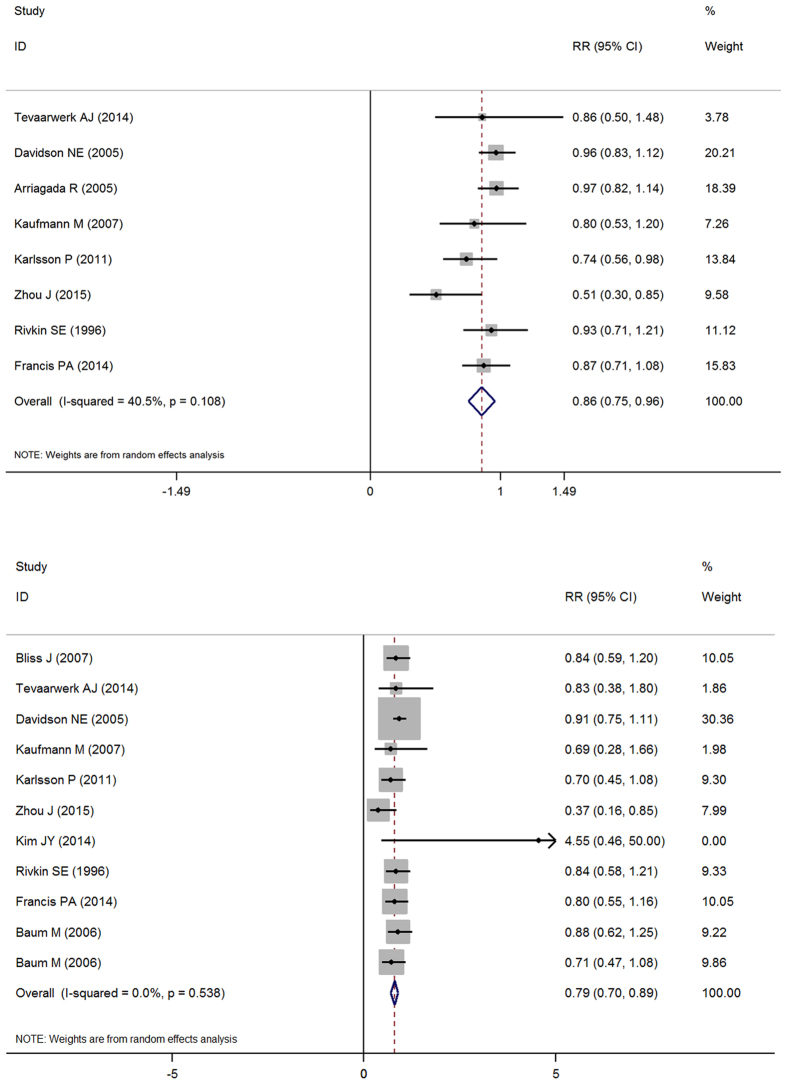


**Figure 4:**
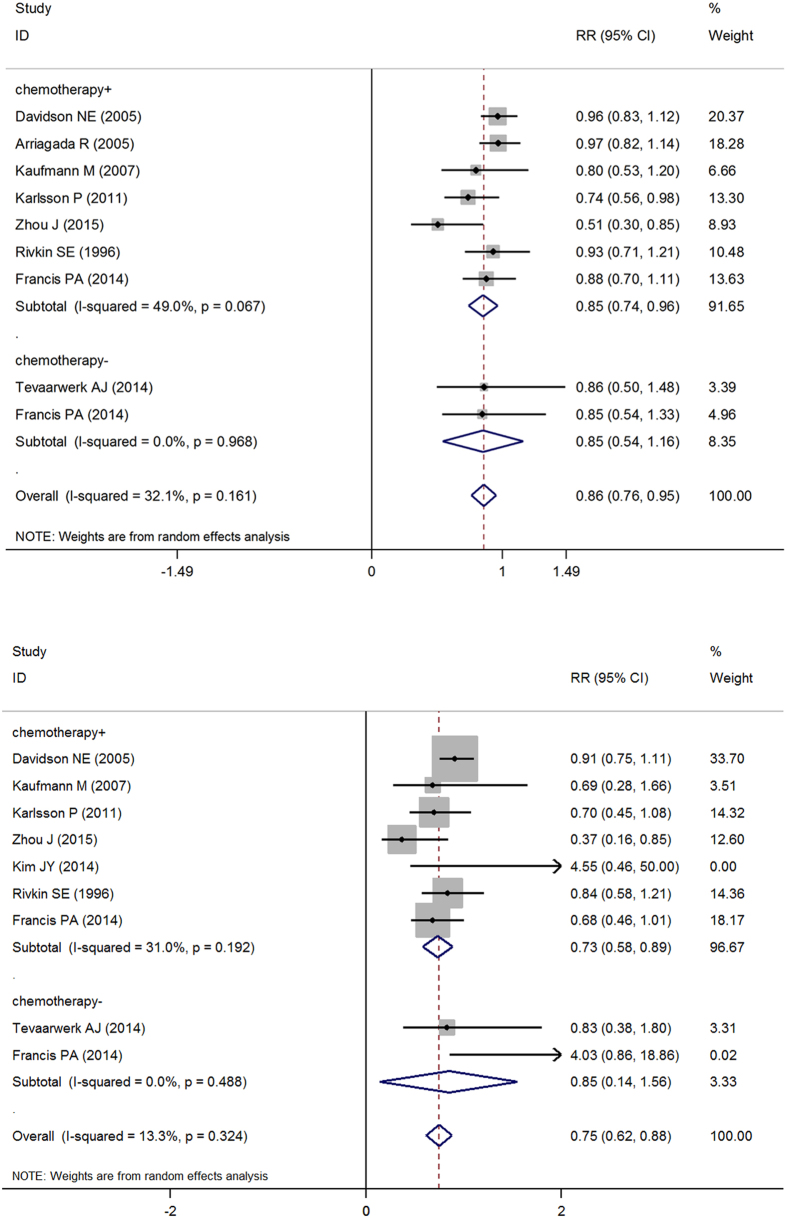


The funnel plots did not suggest the occurrence of publication bias (Supplement Figs 3 and 4).

### Sensitivity analysis

**Figure 5:**
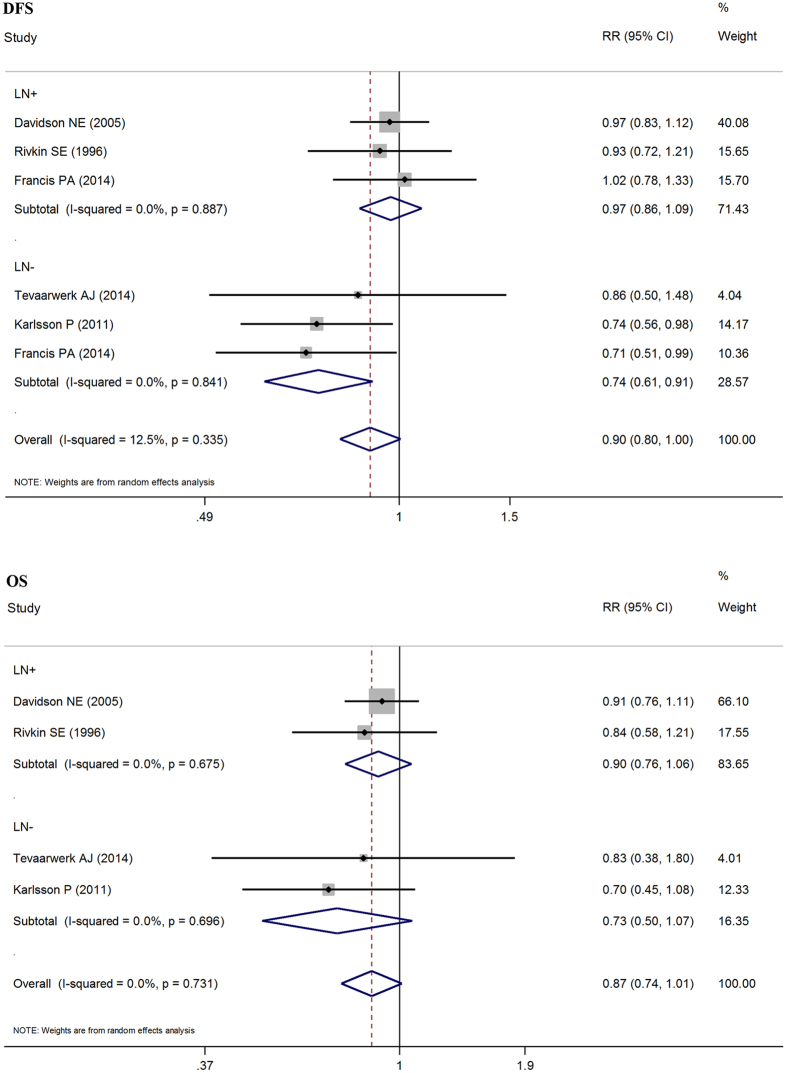


**Figure 6:**
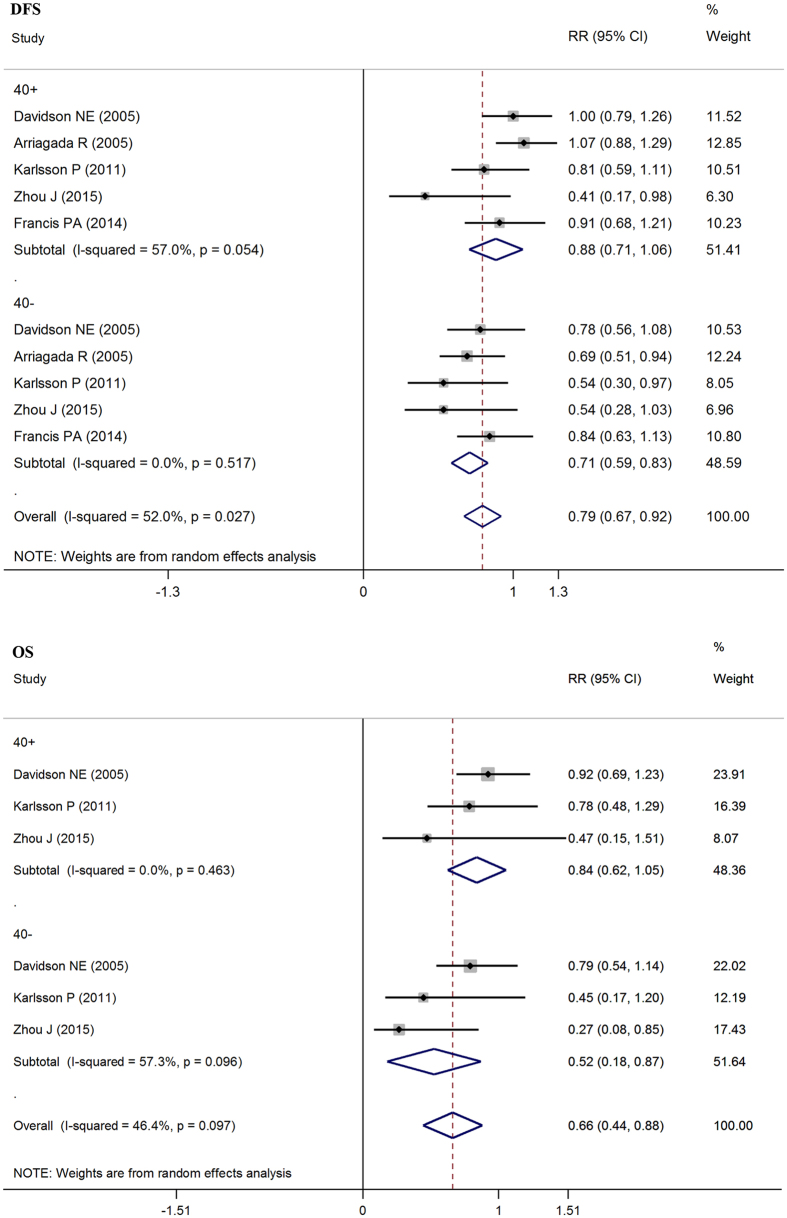


The result is similar to the original one (Supplement Fig. 5).

## Discussion

OFS is an endocrine modifying process with a unique mechanism of action. Its action in the armamentarium against breast cancer therapy remains unclear, although it has been first investigated a century ago.

Here we report a meta-analysis result that OFS can produce survival benefits in premenopausal patients with hormone receptor positive early breast cancer as measured by DFS and OS. The result is similar to two randomized trials, one is published in Cancer Research Treatment of 2015, which showed that Goserelin improved disease-free survival (85.4% vs. 71.6%, p = 0.005), and overall survival (93.5% vs. 83.5%, p = 0.010), the other is IBCSG VIII (DFS: RR = 0.74, 95% CI: 0.56–0.98, OS: RR = 0.70, 95% CI: 0.45–1.08)^19^,^24^. A decline in oestradiol might provide one explanation for the greater efficacy of OFS than no OFS. The Yang H *et al.* randomized controlled clinical trial demonstrated that the addition of goserelin to tamoxifen results in downregulation of estradiol levels compared with tamoxifen alone^28^.

However, statistical significance was observed in two of these studies in DFS and in one of these in OS (Fig. 3). The E3193, INT-0101, FNCLCC, Kim JY 2014, Rivkin SE 1996 all compared chemotherapy with chemotherapy and OFS, not including tamoxifen. Due to no subsequent endocrine therapy, they eventually led to negative results. In the latest large phase III trial (SOFT), the higher risk cohort of patients who remained premenopausal after chemotherapy with tamoxifen plus ovarian suppression resulted in an absolute improvement of 4.5 percentage points as compared with tamoxifen alone^20^. Maybe this is a reason the results showed no statistical significance. However, the IBCSG VIII is the comparison of the same. Through analyzing, we find that the result has statistical significance during 12 years of follow-up (DFS: RR = 0.74, 95% CI: 0.56–0.98 OS: RR = 0.70, 95% CI: 0.45–1.08), but it has no significance during five (5) years of follow-up (DFS: RR = 0.84, 95% CI:0.56–1.26). A meta-analysis shows that different breast cancer subtypes based on ER and HER2 module scores have different recurrent time^29^, and this may be the reason to explain the difference. In the ABC trial and ZIPP trial that compared tamoxifen with tamoxifen plus OFS in which some patients were selected to receive chemotherapy, the result also has no significance. Results of EBCTCG overview indicate that the benefits of ovarian ablation or suppression on relapse-free survival only become apparent after several years and that overall survival gains appear at 8–10 years follow-up^21^. It is possible that benefits of OFS will emerge after further follow-up data have accrued. In E3193 trial compared tamoxifen with tamoxifen plus OFS, it also has no statistical significance, in which the population is low-risk, whose axillary node is negative, and tumors are less than 3 cm. The St Gallen consensus 2015 proposes the low risk premenopausal patients only need to use tamoxifen, which is consistent with the result^30^.

Our data also show that patients gained survival benefits from OFS in the chemotherapy group but not in the no chemotherapy group. The population in no chemotherapy group is low risk, and the patients included are axillary node-negative, with tumors measuring less than 3 cm and hormone receptor positive in the E3193 trial, which remained premenopausal after the completion of adjuvant or neoadjuvant chemotherapy^18^. In the SOFT trial, most of these patients are lymph node negative, with tumors measuring less than 2 cm, and the histological grade was less than 2^20^. In the St Gallen International Expert Consensus on the primary therapy of early breast cancer 2015, it is said that endocrine therapy for premenopausal patients at low risk should comprise tamoxifen for five (5) years, while those at higher risk should be considered for OFS and the substitution of exemestane or tamoxifen^30^. This is consistent with our results. In the chemotherapy group, adding OFS is superior to no OFS. In a retrospective NSABP analysis of 5,849 premenopausal women treated with chemotherapy alone, women younger than 35 years with hormone-sensitive disease had a significantly higher risk of relapse than women who were age 35 years or older, which suggests that chemotherapy alone is insufficient treatment for younger women with hormone-sensitive disease. Our chemotherapy with no OFS group also has some patients who are less than 35 years old^31^. At the same time, in the International Breast Cancer Study Group (IBCSG) VIII, the Subpopulation Treatment Effect Pattern Plot (STEPP) analysis clearly shows an enhancement effect of amenorrhea with CMF followed by goserelin compared with CMF alone at younger ages (<40 years of age) with ER-positive disease^24^,^32^.

Another result analysis shows that patients under 40 years of age would benefit to a greater extent from OFS when compared with those more than 40 years of age. A possible explanation is that chemotherapy alone is insufficient treatment for younger women with hormone-sensitive disease and the use of OFS can contribute to more survival benefit for younger breast cancer patients. In a review published in Cancer Research and Treatment 2015, it was indicated that anthracyclines and alkylating agents commonly used in chemotherapy for breast cancer were reported to induce CIA in 0–46% of patients aged <40 years and in 65–100% of patients aged >40 years^19^. In a retrospective NSABP analysis of 5,849 premenopausal women treated with chemotherapy alone, women younger than 35 years with hormone-sensitive disease had a significantly higher risk of relapse than women who were 35 years or older^31^. It also illustrates that chemotherapy could not satisfy the need of younger breast cancer patients. The other reason is that the OFS plus chemotherapy could enhance the effect of amenorrhea in those treated with chemotherapy alone. The IBCSG VIII trial has meticulously record the amenorrhea rate of patients at younger ages (<40 years of age) with ER-positive disease from chemotherapy, goserelin and chemotherapy plus goserelin^24^. Another reason is that OFS reduced the estradiol level when added to the tamoxifen treatment compared with tamoxifen. A randomized controlled clinical trial demonstrated that the addition of goserelin to tamoxifen results in downregulation of estradiol level^28^.

Another subgroup is lymph node positive patients who do not benefit from our subgroup analysis. In three trials included, two compared chemotherapy plus OFS with chemotherapy alone, not including tamoxifen. The former said that the addition of goserelin to tamoxifen results in downregulation of estradiol levels. It may be a reason to explain the heterogeneity. Even though the SOFT trial adding to the tamoxifen did not obtain the survival benefit, the higher risk cohort of patients who remained premenopausal after chemotherapy with tamoxifen plus ovarian suppression resulted in an absolute improvement of 4.5 percentage points as compared with tamoxifen alone^20^. The high risk factors also need to be considered when using OFS.

Our data also show some toxicity. Adding OFS resulted in increased adverse events, mild or severe hot flashes and hypertension, an observation consistent with the findings of the SOFT, ZIPP and INT0101 trials. These toxicities need to be weighted with any benefit from OFS. At the same time, we also compare the difference in the weight gain and diabetes. A statistical heterogeneity was found, which might be related to some trial features, including differences in the characteristics of the populations and the period of using luteinizing hormone releasing hormone. More clinical trials are needed to explore and improve toxicity data.

This meta-analysis has some limitations. First, even though the populations included are premenopausal, it included two kinds, one of which is premenopausal before treatment and the other which is premenopausal after treatment. Findings of the IBCSG study indicate that LHRH agonists can improve the therapeutic efficacy of patients without CIA. This might have influenced the final result of survival benefit of OFS. The second limitation is in the extent of intervening measures. Only did two of the eleven trials compare tamoxifen plus OFS with tamoxifen in the patients who are still in premenopausal after chemotherapy.A conclusive evaluation of tamoxifen and OFS will require further clinical trials. Third, LHRH was used for different times in various studies. The duration of ovarian function suppression is 2 to 5 years in general. There are no clinical trials to compare the effect of duration of LHRH on the survival benefit, and further research is still needed. Fourth, due to the included trials of this article needing to be published, the different results between the published articles and the unpublished articles may affect the final result. Fifth, in order to execute the inclusion criteria in strict rotation, the articles included are few.

## Methods

### Type of studies and participants

This systematic review included published prospective cohort trials. We included patients who are all hormone receptor positive (positive for estrogen receptor, progesterone receptor, or both) and premenopausal early breast cancer patients. The definition of premenopausal has two layers of meaning, one of which is premenopausal before chemotherapy and the other of which is premenopausal after chemotherapy. Specifically, including a menstrual period within the past six months without prior oophorectomy, or in the case of prior hysterectomy being 55 years or younger with one or both ovaries remaining and a hormone level in the normal premenopausal range^18^. This also includes patients who had regular menstrual cycles or temporary amenorrhea (the resumption of menstruation after at least three months of amenorrhea) after chemotherapy or levels of estradiol follicle-stimulating hormone (FSH) and luteinizing hormone similar to premenopausal levels^19^. Early breast cancer is defined as operable disease and no distant metastasis, so patients included had received a mastectomy and an axillary lymph node dissection, or a sentinel lymph nodes biopsy.

### Types of intervention

Included trials compared OFS with no OFS where OFS was achieved by using luteinizing hormone releasing hormone (LHRH) administered by means of intra-muscular injection every 28 days, bilateral oophorectomy, or bilateral ovarian irradiation^20^. The experimental groups and control groups had the same treatment except for the OFS; including the surgery, chemotherapy and endocrine therapy.

### Outcome measures

The primary outcome of this review was DFS defined as the time from randomization to the diagnosis of recurrent or metastatic disease, DFS was determined for both the OFS group (experimental group) and the no OFS group (control group). The OS was defined as death from any cause at the study endpoint in all included studies. The relative ratio (RR) was the standard of comparison for DFS and OS of the experimental and control groups. The following secondary outcomes were assessed; side-effects that included hot flashes, hypertension, weigh gain, and diabetes.

### Included criteria

The articles those met the following inclusion criteria were adopted for the meta-analysis: 1. about OFS; 2. patients who are all hormone receptor positive (positive for estrogen receptor, progesterone receptor, or both) and premenopausal early breast cancer patients; 3. clinical trials; 4. evaluating DFS and OS of patients.

### Exclusion criteria

This systematic review excluded the relevant articles based on the following: 1. Irrelevant articles which do not discuss the OFS; 2. The patients who have metastasis or local recurrence; 3. Not a clinical trial; 4. Not relevant for the endpoint of the study; 5. Using OFS in each group; 6. the trials in the same population and in different follow-up time, choosing one of them.

### Search methods for study identification

Literature searches were performed using the following databases (last update: August 2015): PUBMED, GOOGLE SCHOLAR, EMBASE, OVID and COCHRANE.

### Selection of studies

We examined all titles and abstracts through GOOGLE SCHOLAR with keywords “LHRH agonist”, “luteinising-hormone-releasing hormone”, and “breast cancer”, the PUBMED search strategy used “Gonadotropin-Releasing Hormone” or “Goserelin”, and “breast neoplasms”, the EMBASE database was searched with phrases “breast tumor”, “hormone therapy”, “premenopause” and “gonadorelin agonist”, the OVID database with the words “breast cancer “, “premenopausal “, “goserelin “ and the COCHRANE database with “breast cancer”,“endocrine therapy” and “Gonadotropin-Releasing Hormone”. We obtained full texts of potentially relevant papers. We also manually search relevant references or clinical trials according to some review articles we recovered. Considerable care was taken to exclude duplicate publications. There are in all 1,317 publications that were reviewed.

### Qualitative assessment

Quality assessment was performed in each of the acceptable studies using the Jadad Quality Assessment Scale for cohort studies^33^ (Table 2).

### Data extraction

Data extraction included participants’ characteristics, such as the name of the clinical tests, first author, date of publication, the number of patients reported, follow-up time, hormone receptor status, the condition of the lymph nodes, intervention details, and outcome.

### Data analysis

Log hazard ratios and their variance were computed separately for each trial and a fixed-effect estimate of the overall log hazard ratio and its variance were calculated with the inverse variance-weighted method. The analysis was done in Stata (version 12) using the meta command. Results are presented as changes in relative ratio with 95% CI.

### Assessment of heterogeneity

Visual inspection of graphs was used to investigate the possibility of statistical heterogeneity. This was supplemented using the I-squared statistic and p values. This provides an estimate of the percentage of variability due to heterogeneity rather than chance alone. Where the I-squared was greater than or equal to 50%, or the p value was less than 0.05, we interpreted this as indicating the presence of a high level of heterogeneity.

### Assessment of publication bias

For the overall outcome, the funnel plot approach was used to investigate publication bias^24^.

### Sensitivity Analysis

We performed the sensitivity analyses through comparing the difference between the result of original data and the one after picking out the results of Zhou J^19^ and Kim JK^25^ which of qualitative assessment are poor.

### Subgroup analyses

The following pre-planned subgroup analyses were carried out; chemotherapy group versus no chemotherapy group, lymph node negative versus lymph node positive, age more than 40 years versus less than 40 years, and side-effects including hot flashes, hypertension, weigh gain, and diabetes.

## Additional Information

**How to cite this article**: Qiu, L. *et al.* Evaluating the Survival Benefit Following Ovarian Function Suppression in Premenopausal Patients with Hormone Receptor Positive Early Breast Cancer. *Sci. Rep.* **6**, 26627; doi: 10.1038/srep26627 (2016).

## Supplementary Material

Supplementary Information

## Figures and Tables

**Table 1:** The characteristic of randomized clinical trials.

SN	Name	Author	Year	Total	HR	LN	Follow-up	Treatment
1	ABC Trial^21^	Bliss J	2007	2144	−/+	−/+	5.9	TA+/−chemotherapy+ofs vs TA+/−chemotherapy
2	E-3193^18^	Tevaarwerk AJ	2014	345	+	−	9.9	TA vs TA +ofs for 5y
3	INT0101(E5188)^16^	Davidson NE	2005	1503	+	+	9.6	6CAF vs 6CAF+gos 5y vs 6CMF+TA+gos
4	FNCLCC^22^	Arriagada R	2005	926	−/+	−/+	9.5	chemotherapy vs chemotherapy+ofs
5	GABG-IV-B-93^23^	Kaufmann M	2007	392	−/+	−/+	4.7	Chemotherapy vs chemotherapy+gos for 2y
6	IBCSG-VIII^24^	Karlsson P	2003	1111	−/+	−	12	gos 2y vs CMFvs CMF+gos1.5y
7	Zhou J^19^	Zhou J	2015	353	+	−/+	5	chemotherapy+gos+TA for 31m vs chemotherapy+TA
8	Kim JY^25^	Kim JY	2014	436	−/+	−/+	4	chemotherapy+gos vs chemotherapy
9	Rivkin SE^26^	Rivkin SE	1996	340	+	+	7.7	CMFVP vs CMFVP+ovariectomy
10	SOFT^20^	Francis PA	2014	2032	+	−/+	5.5	chemotherapy+TA vs chemotherapy+TA +ofs for 5y
11	ZIPP^27^	Bauma M	2005	2710	−/+	−/+	5.5	TA+/−chemotherapy vs TA+/−chemotherapy+ofs
								ofs+/−chemotherapy vs control+/−chemotherapy

HR = hormone receptor; LN = lymph nodes; TA = tamoxifen; vs = versus; ofs = ovarian function suppression or ablation; y = years; gos = goserelin; CAF = cyclophosphamide, doxorubicin, and fluorouracil; CMF =  cyclophosphamide, methotrexate and fluorouracil; VP = vincristine and prednisone.

**Table 2:** The quality evaluation of included clinical trials by Jadad scale.

SN	Name	random	Allocation concealment	blindness	result	score
1	ABC Trial^21^	1	0	0	1	2
2	E-3193^18^	2	0	0	1	3
3	INT0101(E5188)^16^	1	0	0	1	2
4	FNCLCC^22^	2	0	0	1	3
5	GABG-IV-B-93^23^	1	0	0	1	2
6	IBCSG-VIII^24^	1	0	0	1	2
7	Zhou J^19^	0	0	0	1	1
8	Kim JY^25^	0	0	0	0	0
9	Rivkin SE^26^	1	0	0	1	2
10	SOFT^20^	2	0	0	1	3
11	ZIPP^27^	1	0	0	1	2
