# Supplementary material for: Evaluating the Survival Benefit Following Ovarian Function Suppression in Premenopausal Patients with Hormone Receptor Positive Early Breast Cancer
Source: Sci Rep. 2016 May 27;6:26627. doi: 10.1038/srep26627 (PMC4882507; doi:10.1038/srep26627)

Evaluating the Survival Benefit Following Ovarian Function Suppression in Premenopausal Patients with Hormone Receptor Positive Early Breast Cancer

Lin Qiu a+, Fangmeng Fu b+, Meng Huang c+, Yuxiang Lin b, Yazhen chen a, Minyan Chen b, Chuan Wang b*

a: Union Hospital School, Fujian Medical University, Fuzhou, China

b. Department of Breast Surgery, Affiliated Union Hospital of Fujian Medical University, Fuzhou, China

c. Fujian Center for Disease Control and Prevention, China

+These co-first authors contributed equally to this work

*Corresponding Author: Chuan Wang, Department of Breast Surgery, Affiliated Union Hospital of Fujian Medical University, 29 Xinquan Road, Gulou District, Fuzhou 350001, China.

Phone: 8613515020716, Fax: 0591-88261132, E-mail: chuanwang1968@yahoo.com.

1.the comparison of side-effect of OFS

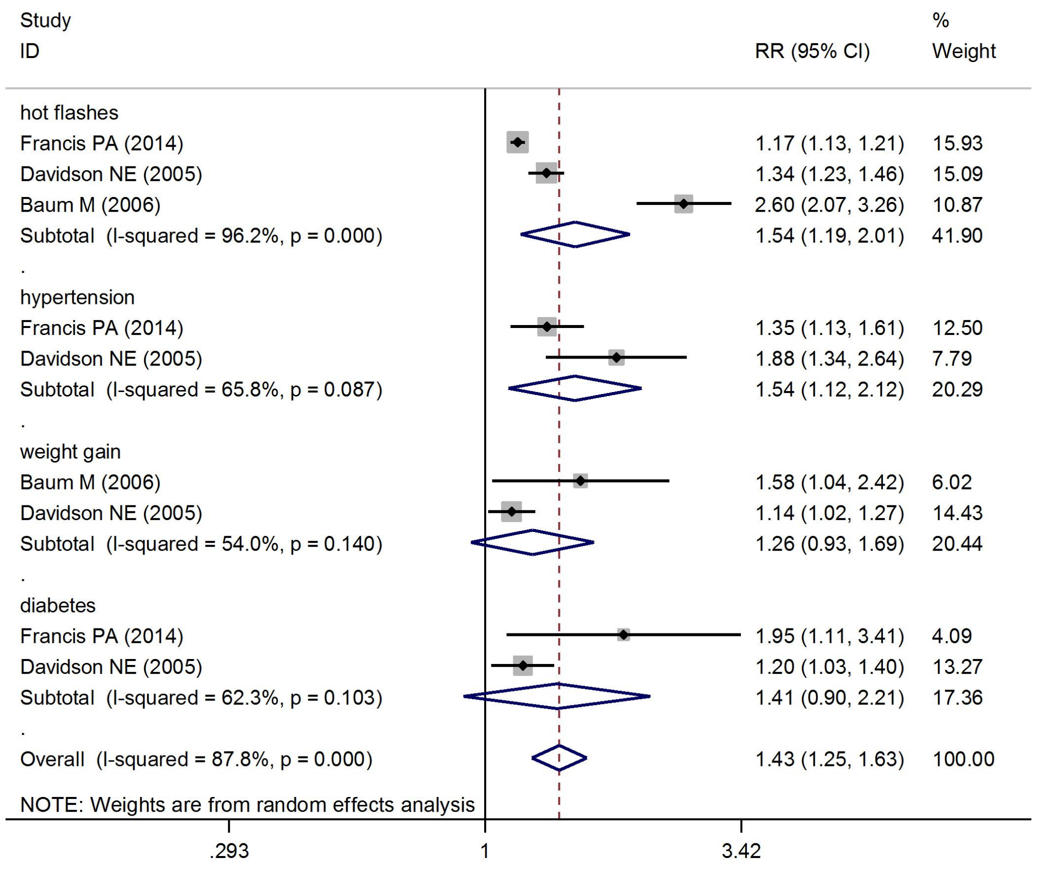

2. the comparison of side-effect of OFS on more than grade 3

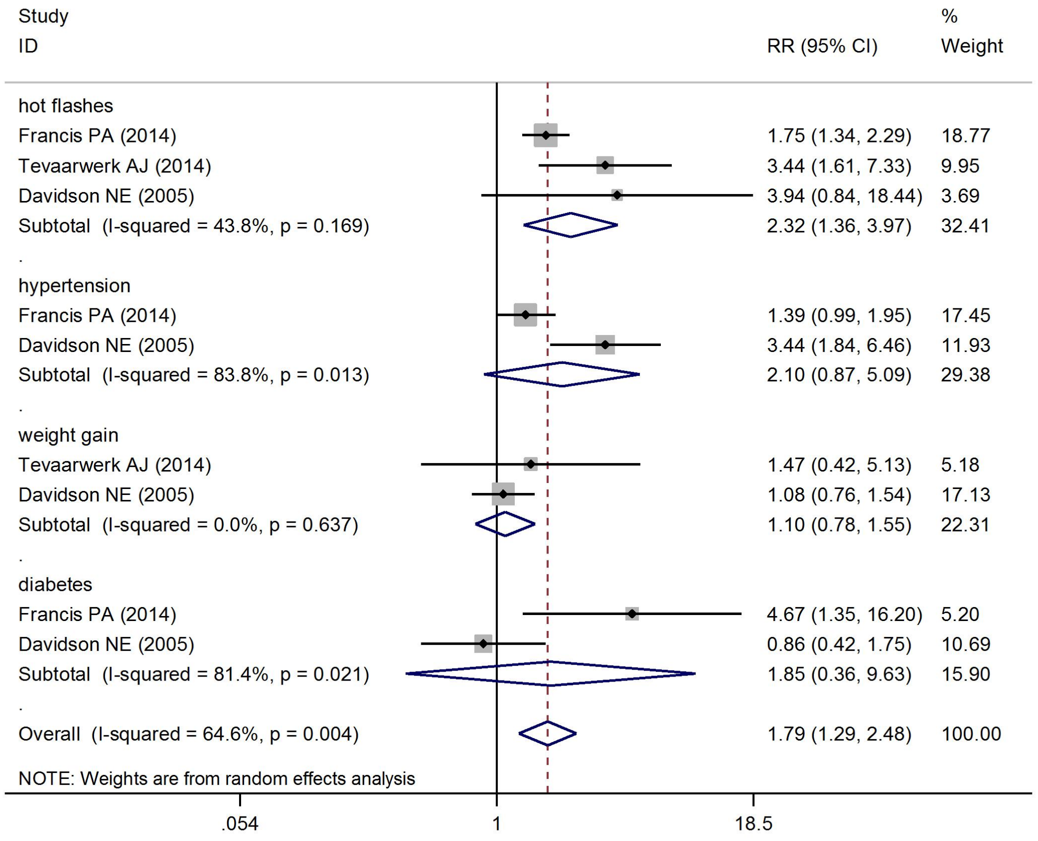

3.Funnel plot of effect OFS in DFS for the included studies

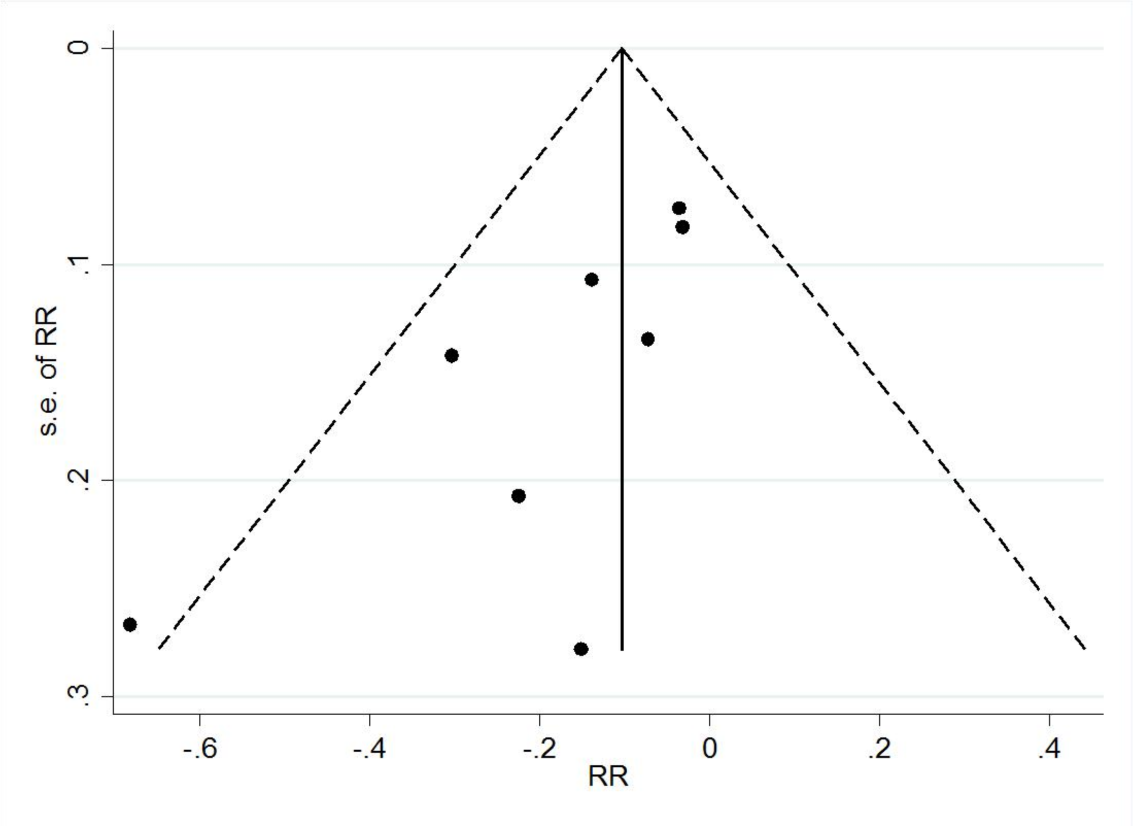

4.Funnel plot of effect OFS in OS for the included studies

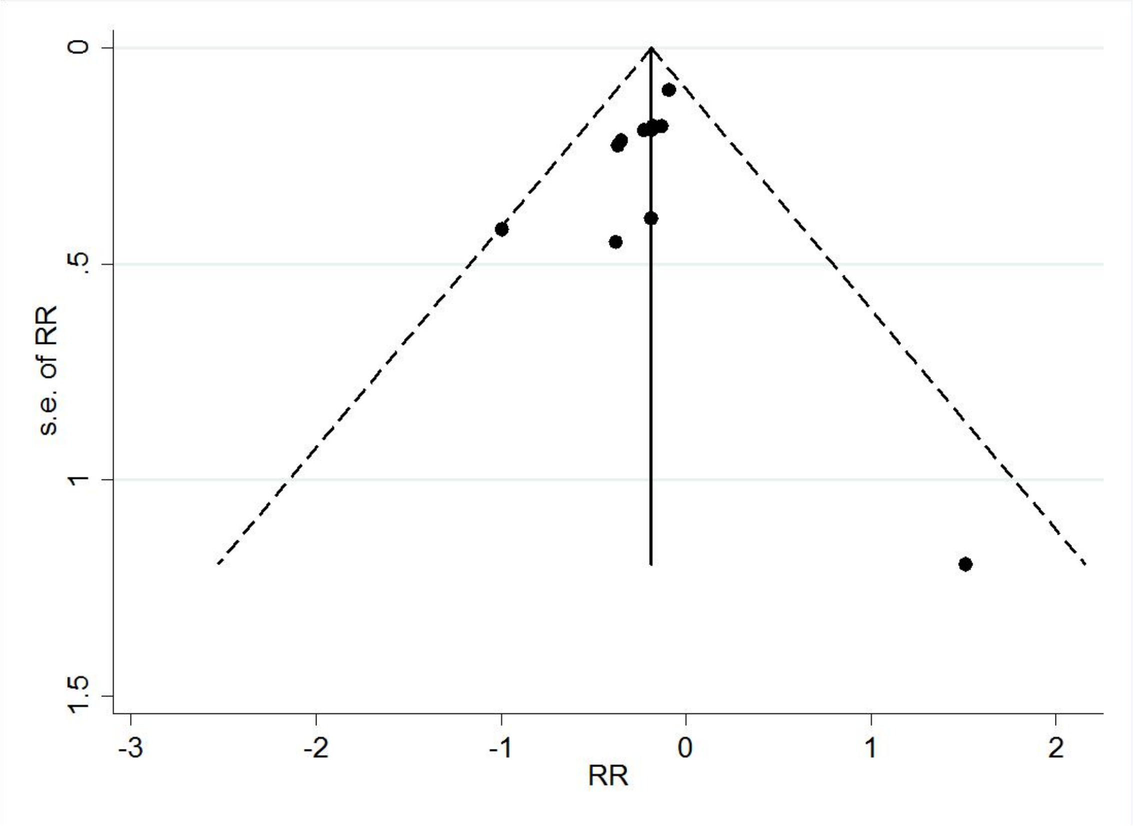

5.A forest plot showing the difference of total DFS and OS in using OFS or not after picking out

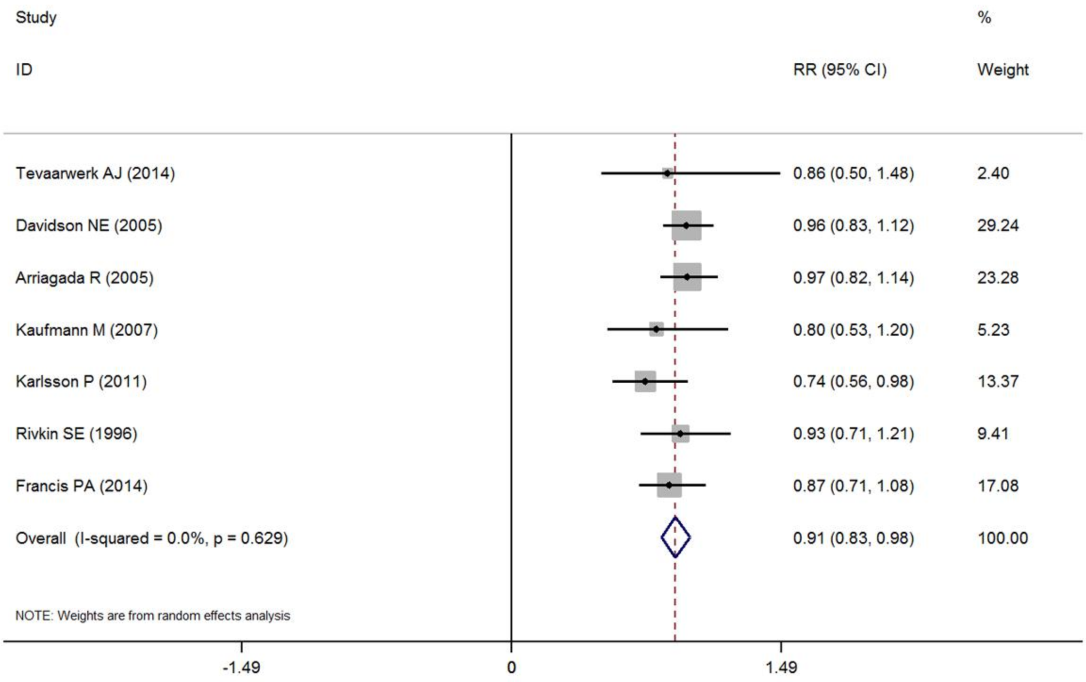

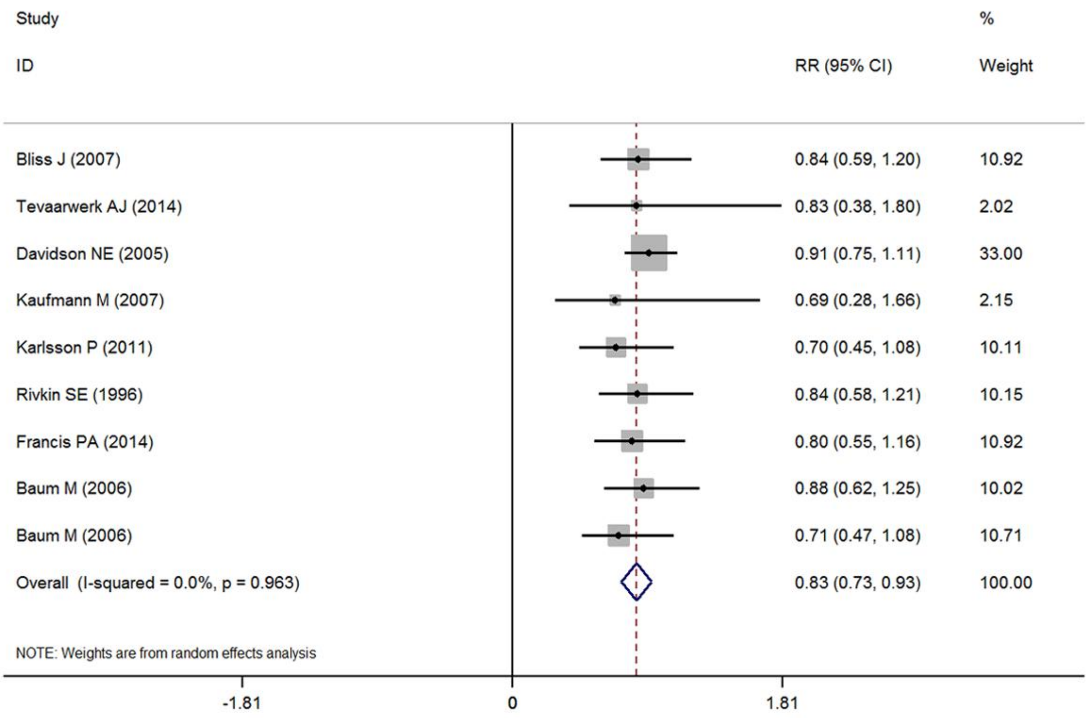

Supplement: Supplementary Information [file srep26627-s1.pdf]
